# Sensorimotor Manipulations of the Balance Control Loop–Beyond Imposed External Perturbations

**DOI:** 10.3389/fneur.2018.00899

**Published:** 2018-10-26

**Authors:** Brandon G. Rasman, Patrick A. Forbes, Romain Tisserand, Jean-Sébastien Blouin

**Affiliations:** ^1^Department of Neuroscience, Erasmus Medical Center, Rotterdam, Netherlands; ^2^School of Kinesiology, University of British Columbia, Vancouver, BC, Canada; ^3^Department of Biomechanical Engineering, Faculty of Mechanical, Maritime and Materials Engineering, Delft University of Technology, Delft, Netherlands; ^4^Djavad Mowafaghian Center for Brain Health, University of British Columbia, Vancouver, BC, Canada; ^5^Institute for Computing, Information and Cognitive Systems, University of British Columbia, Vancouver, BC, Canada

**Keywords:** imposed perturbations, ongoing human in the loop manipulations, balance control, quiet standing, robotics, sensory stimulation

## Abstract

Standing balance relies on the integration of multiple sensory inputs to generate the motor commands required to stand. Mechanical and sensory perturbations elicit compensatory postural responses that are interpreted as a window into the sensorimotor processing involved in balance control. Popular methods involve imposed external perturbations that disrupt the control of quiet stance. Although these approaches provide critical information on how the balance system responds to external disturbances, the control mechanisms involved in correcting for these errors may differ from those responsible for the regulation of quiet standing. Alternative approaches use manipulations of the balance control loop to alter the relationship between sensory and motor cues. Coupled with imposed perturbations, these manipulations of the balance control loop provide unique opportunities to reveal how sensory and motor signals are integrated to control the upright body. In this review, we first explore imposed perturbation approaches that have been used to investigate the neural control of standing balance. We emphasize imposed perturbations that only elicit balance responses when the disturbing stimuli are relevant to the balance task. Next, we highlight manipulations of the balance control loop that, when carefully implemented, replicate and/or alter the sensorimotor dynamics of quiet standing. We further describe how manipulations of the balance control loop can be used in combination with imposed perturbations to characterize mechanistic principles underlying the control of standing balance. We propose that recent developments in the use of robotics and sensory manipulations will continue to enable new possibilities for simulating and/or altering the sensorimotor control of standing beyond compensatory responses to imposed external perturbations.

## Introduction

Our ability to stand upright requires accurate estimation about the orientation of the body with respect to gravity as well as the relative relationships between body segments. These estimates are formed through multisensory integration of information arising from visual, vestibular, somatosensory and auditory sensory systems. Imposed perturbations of the sensory/motor systems and manipulations of the balance control loop provide methods of disrupting and/or modifying the balance controller. These approaches, however, differ. Imposed perturbations (transient or continuous) evoke external error signal inputs while manipulations of the balance control loop are designed to modify the sensorimotor relationships required to control quiet stance. Both approaches have proven critical in unraveling fundamental sensorimotor principles underlying standing balance. In this review, we explore perturbation and manipulation approaches used to probe the balance system. We first provide an overview of the sensorimotor and mechanical characteristics that are relevant for the control of standing balance. Then, we discuss imposed external perturbations that have enabled researchers to investigate how the balance system responds to these unexpected disturbances. Here, we operationally define imposed perturbations as methods which disrupt quiet standing behavior and represent external error signals for the balance system. As such, the parameters of the imposed external perturbations are designed exclusively by the experimenter. We subsequently present manipulations of the balance control loop that can be implemented to alter sensory feedback and/or their relationships with motor outputs during the ongoing control of quiet standing balance. Importantly, although these techniques can involve physical and/or sensory alterations, we define them as manipulations (rather than perturbations) as they are designed to modify relationships within the balance control loop such that their effects are a function of the action of the subject (i.e. human in the loop manipulations). Finally, we emphasize how manipulations of the balance control loop altering ongoing feedback can be combined with imposed perturbations to reveal sensorimotor principles of standing balance. Throughout this review, we prioritize information gained from experimental approaches applied to healthy human volunteers. Where appropriate, we relate these findings to observations gathered from clinical populations (e.g., persons with vestibular loss), whose behavior may complement our insight into the control of standing balance.

## Sensorimotor and mechanical aspects of standing balance

The upright bipedal posture adopted for standing balance is mechanically unstable. When the vertical projection of the whole-body deviates from the ankle joint center of rotation, gravity acting on the body increases the magnitude of the toppling torque and must be compensated by active and passive forces. Consequently, although standing may be referred to as quiet stance or static balance, the acceleration of the whole-body center of mass is constantly varying in three-dimensional space. The mechanics of standing balance involve both large and fine adjustments used to stabilize the whole-body and the relative orientation of body segments (1–4). The mechanics of standing balance are often simplified by assuming that movement only occurs around a limited number of joints. In the anteroposterior direction, the standing body is commonly represented using a single-link inverted pendulum model where whole-body movements occur mainly around the ankle joints (5–8). In the mediolateral direction, an inverted pendulum with dual links (i.e., both limbs) has been proposed (9–11), with whole-body motion occurring around both the ankle and hip joints. The differential equation of an inverted pendulum is therefore used to describe the relationship between the net torque and whole-body angle when a person stands; explaining how body inertia mechanically filters muscle activation during standing, resulting in low frequency movements of the whole-body (typically below 0.5 Hz for quiet standing sway) (8, 12–14).

**Figure 1:**
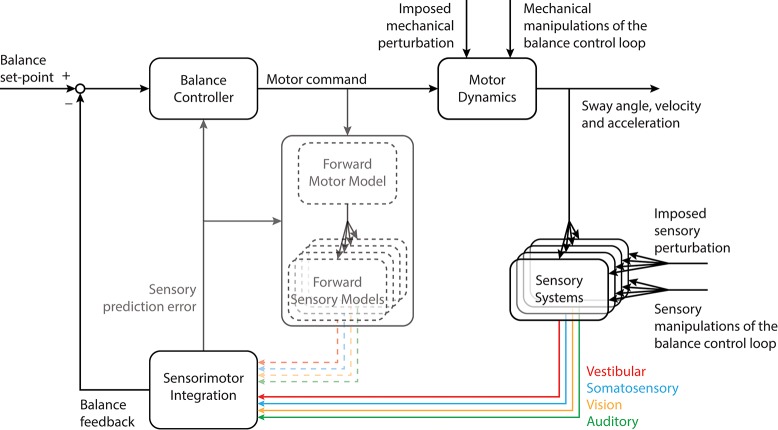
Schematic of the balance control loop depicting the relationship between motor command, sensory feedback and multisensory integration. Sensory cues convey information about the external world and the body's orientation within it. This information is integrated with motor commands to estimate sensory prediction errors. Because these aspects of the balance control loop are not the focus of this review, they have been grayed out in the figure [see (24) for a more detailed exploration of these principles]. Mechanical and sensory inputs to the control loop illustrate a conceptual representation of imposed perturbations or manipulations of the balance control loop applied to understand the control of standing balance. Details on the implementation of imposed perturbations or manipulations of the balance control loop are presented in Figure 2. Portions of this figure were adapted from Forbes et al. (24).

Given that forces are developed when musculoskeletal tissues are deformed, it has been proposed that tonic muscle activity may be sufficient to maintain standing balance passively (11, 15). For balancing along the anteroposterior direction, however, the toppling gravito-inertial torque associated with whole-body movements exceeds the intrinsic stabilizing torque developed by the viscoelastic forces during deformation of ankle tissues (16, 17). Consequently, active neural control of the ankle and hip muscles (as well as those acting at other joints) is required to stabilize the body and modulate the net forces and torques delivered through the feet onto the support surface (18–23). The active maintenance of standing balance involves a sensorimotor control loop that detects body orientation/motion and generates the stabilizing forces and torques required to remain upright (Figure 1). Information regarding the orientation of the body with respect to gravity and the relative relationship between body segments is provided by integrating multiple cues from sensors located throughout the body. In the following paragraphs, we describe briefly the balance-relevant sensory code provided by these sensors as they relate to the frequency characteristics of standing balance.

### Balance-relevant sensory code

Sensory inputs from the visual, vestibular, somatosensory and auditory systems all contribute to the control of standing balance. The information provided by individual sensory cues is shaped by the dynamics of each sensor and the coordinate system in which they are referenced [for a review of sensory dynamics related to standing balance, see (24)]. To be relevant for standing balance, a sensor must be capable of encoding frequencies up to and beyond those comprising the dynamics of the standing body; i.e., the dynamics of a sensor must be greater than the actuator, which must be greater than the mechanical system being controlled (25–27). Therefore, sensors that primarily encode low frequency (and static) information may be more likely to contribute to the low frequency control of quiet standing balance whereas those encoding higher frequencies may be more helpful in responding to imposed external perturbations.

The somatosensory system refers to a group of receptors found throughout the muscles, joints, and skin of the body. Several of these mechanoreceptors relay position and motion cues referenced to the body and its different segments, also known as proprioception [(28–33); for a comprehensive review, see (34)]. Collectively, muscle and joint receptors encode static and dynamic joint angle and/or muscle force. Although cutaneous receptors may also encode joint angle (35), those located in the glabrous skin of the foot sole act as an interface between the external world and the body. They can sense contact forces and texture of the support surface that may be used for standing balance (36, 37). The visual system encodes cues referenced to the external world derived from our field of view. From visual inflow, motion signals of the surrounding world (object-motion) and of the body within the world (self-motion) are extracted and provide cues to stabilize the upright body (38). The accessory optic system (a series of nuclei in the midbrain with efferent connections to the brainstem and cerebellum) likely plays an important role for balance control given its preference for low frequency stimuli and interaction with vestibular inputs (39, 40). Visual signals further provide cues on the spatial orientation of objects in our surroundings that may be used for controlling posture and responding to disturbances (41, 42). The vestibular end organs, which are fixed within the inner ears, sense three dimensional orientation and inertial cues of the head-in-space (43). Two subtypes of end organs, the otoliths and the semicircular canals, allow the vestibular apparatus to encode translational and angular motion, respectively (44). Because otoliths also encode head orientation relative to gravity, the distinction between head orientation with respect to gravity and head acceleration signals can be achieved by the integration of otolith and canal cues along with visual and somatogravic ones (45–49). Hence, information derived from the peripheral vestibular apparatus provides important cues needed for the control of standing balance. The auditory system, often overlooked for its role in balance control, is situated alongside the vestibular apparatus in the inner ear. Auditory cues can be used for spatial localization of the head-in-space and produce illusions of self-motion (50, 51), most prominently in the absence of vision (52). When standing, stationary sound cues that are coherent with other sensory signals of balance allow subjects to construct spatial auditory maps that improve postural stability [see review by Campos et al. (53)].

Various imposed stimuli or sensory manipulations of the balance control loop can be used to investigate the role of sensory cues in balance control. In the following sections, we first describe imposed external perturbations that have been used to study the reactive control of standing balance. We emphasize that stimuli of this type evoke compensatory postural responses to external disturbances. Therefore, a particular focus is put on stimuli that specifically target balance control as opposed to methods that evoke responses irrespective of the need to balance upright (e.g., stretch reflexes). We subsequently present and propose methods that alter the ongoing control of quiet standing balance in order to assess the organization and potential adaptability of the neural control of standing balance.

## Imposed external perturbations to characterize standing balance

**Figure 2:**
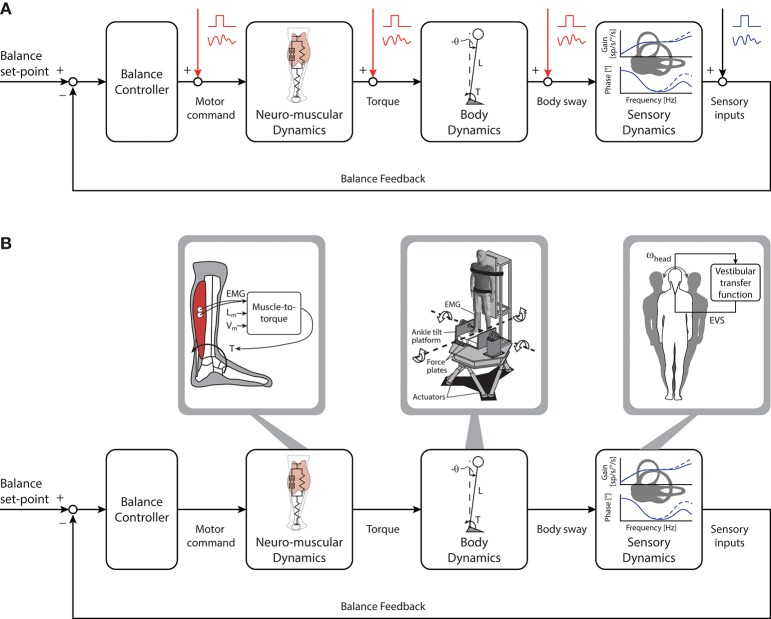
Imposed perturbations and manipulations of the balance control loop. **(A)**: Mechanical and/or sensory perturbations can be imposed at the various stages of the control loop to produce external error signals independent from the ongoing control of quiet standing. Imposed perturbations can be delivered as discrete (e.g., square wave signal) or continuous (oscillatory signal) disturbances to evoke compensatory postural responses. **(B)** Manipulations of the balance control loop aim to simulate and/or modify the relationship between sensory and motor cues of ongoing balance control (i.e. human in the loop manipulations). These manipulations can be used to mimic or alter the dynamics of different components of the balance system through the use of robotic systems and sensory stimulation techniques. Transfer functions characterizing muscle contraction (electromyography [EMG], muscle length [L_m_] and muscle velocity [V_m_]) to torque output can be used to manipulate the ongoing effect of motor command (left). Similarly, a robotic balance simulator can be used to mimic and manipulate balance mechanics (middle). Torque delivered by the subject is used to control platform motion: this places the subject in-the-loop and allows for ongoing manipulation of standing balance. In addition, manipulations to sensor dynamics can be achieved, for example, by using instantaneous head velocity and transfer functions of the vestibular system to deliver an electrical vestibular stimulus that modulates the ongoing vestibular afferent firing rates (right). ω_head_, head rotational velocity; EVS, electrical vestibular stimulation.

Imposed perturbations have been applied extensively to assess the control of standing balance. These perturbations are often designed by experimenters to be similar to disturbances experienced during daily activities (e.g., standing on a bus that suddenly accelerates) and can have a range of amplitudes, velocities and/or accelerations. Carefully applied perturbations have been used to reveal important aspects of standing balance. For example, using imposed external perturbations researchers have estimated the passive and active mechanisms underlying standing balance and revealed how error signals are integrated and transformed to maintain upright stance (see subsections below). A point to consider, however, is that imposed perturbations represent an external error signal that is independent from the quiet standing balancing task (see Figure 2A). Quiet standing balance behaviors can be described using numerical models that also characterize responses to imposed perturbations (54, 55), but it cannot be assumed that the neural processes involved in these two scenarios are identical. Consequently, perturbations imposed on standing participants inform a researcher on how individuals respond to an external disturbance as opposed to how they integrate and combine multisensory cues to maintain quiet stance. Specifically, imposed perturbations may evoke responses originating from sensory cues activated by the perturbation that may not contribute to the control of quiet upright stance. Furthermore, it is currently not possible to estimate the contributions of ongoing sensory feedback involved in maintaining quiet standing balance by introducing additional sensory inputs through imposed perturbations [see (56) for locomotor analogy]. Nevertheless, there are certain imposed perturbations that only evoke whole-body responses when participants are engaged in standing balance and these may reveal fundamental principles underlying its control. We will discuss these different approaches in the following paragraphs but the reader is invited to consult (57–59) for comprehensive reviews of imposed perturbation approaches.

### Mechanical perturbations

A wide variety of mechanical perturbations have been used to study compensatory responses during standing balance. Popular approaches include rotating (60–63) or translating (64–67) the support surface of standing subjects, while others use forces or torques applied to specific points on the body (68–70). When applied as discrete physical perturbations to standing participants, mechanical perturbations evoke stereotypical transient muscle and whole-body responses (71–76). To align better with the continuous control of standing balance and to characterize muscle and whole-body responses to ongoing disturbances, other researchers have used prolonged mechanical oscillations to study standing balance (20, 77–79). Using specific perturbation frequencies and magnitudes, the relationship between oscillatory perturbations and muscle/postural responses can be estimated (80–82). Coupled with sensorimotor modeling, the input/output estimates from prolonged perturbations can reveal fundamental properties of upright stance such as stiffness, damping and time delays of the balance control loop. In animal models, mechanical support surface perturbation approaches have also led to the characterization of synergistic muscle responses in balance control (83–86). Coordinated patterns of muscle activity (i.e. “synergies” or “motor modules”) are thought to be flexibly combined by the nervous system to facilitate functional motor control, and account for spatial, temporal and postural strategy variability in human responses to multidirectional imposed perturbations (87, 88). Mechanical perturbations can also be applied to perturb somatosensory cues of motion without physically moving the whole-body or its support surface. For instance, in “light touch” experiments, perturbations are provided through motion of an external reference that a subject is in contact with (often with a finger) that does not provide mechanical support (89–91). Recently, Asslander et al. (92) perturbed the touch surface that subjects contacted with a finger at different positions with respect to their body. The authors proposed that the brain transforms sensory information derived from light touch into a reference frame for standing balance by estimating the distance between the whole-body center of mass and the finger.

Mechanical stimuli can also be applied to activate cutaneous or muscle receptors. For example, vibration stimuli can be delivered at the foot soles or muscle tendons and adjusted (amplitude and frequency) to elicit responses in cutaneous (primarily fast-adapting) and muscle spindle (primarily Ia) afferents (93–95). When applied to standing participants, these stimuli evoke well-defined and direction-specific whole-body and muscle responses (96–99). Simultaneous vibration of cutaneous and muscle receptors elicits body tilts equal to the vector summation of individual responses (100), suggesting a linear combination of these specific stimuli. However, vibration and stretch stimuli are unspecific to balance control because they can elicit muscle responses in participants not maintaining standing balance (101–103). Consequently, it is not clear what (if any) principles specific to the control of quiet standing can be gained from mechanical vibrations targeting muscle(s) or cutaneous receptors.

### Visual perturbations

Visual perturbations can induce illusions of self-motion (i.e., vection) because retinal signals encode motion of the body and/or the environment. The brain must disambiguate these visual signals in order to control standing balance. For example, when standing on an idle train and viewing another train moving slowly, a perception that your train is moving may emerge. Researchers have exploited this ambiguity to investigate the role of visual cues on postural orientation and control of standing balance by imposing discrete translation or rotary visual perturbations (e.g., movements of the walls within a room or projected image). Standing participants exhibit well-defined compensatory balance responses (and illusions of self-motion) to discrete visual perturbations (104–109). The whole-body responses occur in the same direction as the visual motion (104, 110, 111). One explanation for this response is that the imposed visual stimuli are partially interpreted as a consequence of body motion. Hence, when the visual surround moves backwards (i.e., toward a subject), the balance system interprets the perturbation as self-motion in a forward direction which is corrected by leaning backwards. Consequently, visual perturbations provide a window into how visual signals of self-motion contribute to the control of standing balance. Visually-induced balance responses decrease as the amplitude of visual motion increases (20, 112–115). Dokka et al. (114) proposed that because slow visual signals of whole-body motion are more probable than faster motion, the slower visual signals are more likely to be interpreted as originating from self-motion. Day et al. (115) further reported a later visually-evoked balance response (~0.7 s latency) that increases with stimulus velocity. They suggested that the later visually-evoked balance response is related to the alignment of the body to the erroneous estimate of gravity, an estimate that is biased by a prolonged stimulus of visual motion (107, 110, 115). This concept is reminiscent of the multisensory integration processes required to estimate the orientation of gravity from the otolith signals that can lead to an erroneous interpretation of translation (49, 116–119). It further highlights the usefulness of visual perturbations to explore and reveal physiological principles underlying the control of standing balance.

### Vestibular perturbations

Natural activation of the vestibular system requires movements of the head-in-space. Imposed head movements to examine the role of vestibular inputs on standing balance, however, have a limited use because head motion typically results in concomitant activation of other sensory signals. An isolated vestibular perturbation can be achieved by delivering electrical vestibular stimuli (EVS) through electrodes applied over the mastoid processes (assuming subjects keep their eyes closed). Application of such electrical stimuli modulates the activity of all vestibular afferents (increasing firing rates of all afferents under the cathodal electrode and decreasing under the anodal electrode), without having to move the head in space (120–124). Based on the anatomy and physiology of the vestibular system, bilateral binaural EVS is assumed to generate a vestibular error signal of head roll velocity around an axis pointing posterior and ~18° up from Reid's plane (125–128). Although EVS represents a non-physiological stimulus (i.e., activation of all vestibular afferents), responses elicited by EVS are only present in appendicular muscles when subjects are actively engaged in the task of balancing the whole-body (129–131). Hence, EVS can be used to investigate the vestibular control of balance and how vestibular signals are integrated, processed and relied upon for balance control [see reviews, (24, 125)]. We note, however, that this task dependency is not a ubiquitous feature because EVS evokes vestibulocollic reflexes in neck muscles even when the head and body are fully supported (132).

In standing participants, EVS evokes an unexpected vestibular error signal that requires a compensatory balance response to maintain an upright posture (125, 129, 133). The EVS-evoked error signal of head motion is head-referenced, such that its influence on standing balance depends on the orientation of the head with respect to the feet. Consequently, vestibular-evoked muscle and whole-body balance responses are spatially transformed based on head orientation with respect to the feet (134–137). This indicates that the whole-body responses evoked by an isolated vestibular perturbation (EVS) involve multisensory integration of information related to head-on-feet posture (e.g., via proprioceptive inputs) with vestibular cues of motion. Furthermore, the direction of the vestibular-evoked balance responses is influenced by body stability, whereby muscle and balance responses evoked by EVS are larger in the direction where postural stability is reduced (138, 139). This directional modulation of the vestibular-evoked balance responses based on balance stability without changes in sensory feedback may confound conclusions regarding sensory up-weighting of vestibular signals associated with experimental changes in sensory information (e.g., sway referencing or closing the eyes). This is because altering sensory information while balancing may decrease postural stability (i.e., increase sway), making it difficult to attribute the modulation of vestibular-evoked responses to changes in relative sensory information or changes in postural stability and upright position (140). Finally, the task-dependent characteristics of vestibular-evoked balance responses further suggest that they are not indicative of simple reflex arcs but instead reflect organized balance responses involving the integration of multiple sensory and motor cues (139, 141, 142).

## Sensorimotor manipulations targeting the ongoing control of standing balance

**Figure 3:**
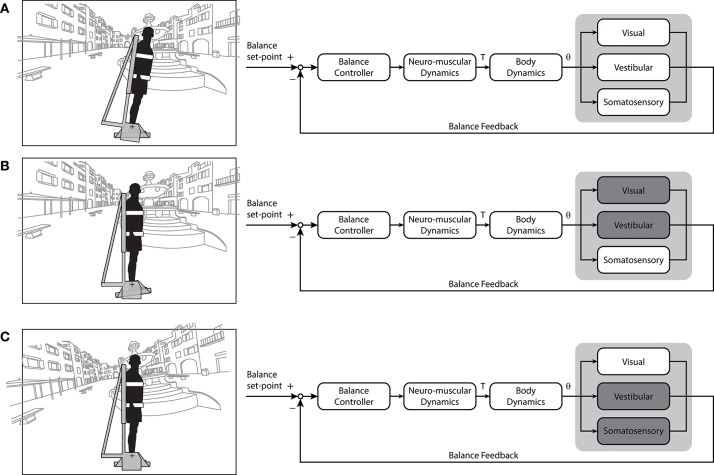
Block diagrams of the varying sensory cue combinations that can be simulated using robotic balance platforms or mechanical devices. **(A)** Normal standing balance conditions where cues from visual, vestibular and somatosensory signals contribute to upright stance. Under these conditions, the foot is stationary, the whole-body moves and the head is moving relative to the visual scene. **(B)** A somatosensory-only balance condition. Subject's head and body are stationary in space in front of a stationary visual surround while the feet rotate, requiring subjects to balance a simulated inverted pendulum that mimics the body's mechanics with movement limited to their ankle joints. **(C)** A vision-only balance condition. Subjects are stationary in space while the visual scene is moving relative to the head, resulting in a balancing task that provides mostly balance-relevant visual cues. This condition was used here to re-examine the potential for standing subjects to use visual cues of motion (see Figure 4). Additional sensory cues (e.g. auditory) and other cue combinations could be considered. For example, by coupling simulated head motion with an electrical vestibular stimulus (see Figure 2B) it may be possible to provide dynamic vestibular cues of standing without actual motion. T, ankle torque; θ, inverted pendulum angle. Portions of this figure were adapted from Shepherd (143).

As discussed in section Imposed External Perturbations to Characterize Standing Balance, imposed perturbations enable the identification and modeling of fundamental principles underlying standing balance. But these approaches must be interpreted within the framework of disturbances external to ongoing control of quiet standing. An alternative approach involves continuous sensory and/or mechanical manipulations of the balance control loop aimed at simulating or modifying the ongoing control of quiet standing balance (see Figures 2B, 3). In other words, these manipulations are designed to modify feedback relationships within the balance control loop such that their effects are a function of the action of the subject (i.e. human in the loop manipulations). In addition, they must carefully match the dynamics of the sensory, motor and mechanical systems involved in standing balance, often requiring detailed knowledge of the neural code to be mimicked or elaborated by devices to induce these manipulations. Here, we review sensory and mechanical manipulations of the balance control loops that allow participants to experience controlled aspects of standing balance or altered sensorimotor conditions. Specifically, we discuss how replicating the sensors dynamics of standing balance can reveal how a specific cue is integrated and processed to maintain upright stability. In addition, we draw parallels between sensorimotor manipulations and specific clinical populations who can balance in the absence of specific sensory feedback cues (e.g., large-fiber sensor neuropathy or vestibular-loss). Where appropriate, we discuss limitations of sensorimotor manipulations and identify where additional work is needed.

### Somatosensory cues

The role of somatosensory cues in the control of standing balance can be partially investigated using ongoing mechanical manipulation of the support surface. Continuous manipulation of the support surface can be adjusted based on the participants' torque production and whole-body postural oscillations to minimize ankle plantar- and dorsi-flexor movements. This sway-referencing of the support surface reduces the contribution of lower limb receptors encoding ankle angle to the control of standing. The increase of whole-body oscillations observed under this condition has been interpreted as supporting the role of ankle somatoreceptors in the control of standing balance (144–147). Reports from the clinical literature add support to the importance of somatoreceptors in upright postural control: patients with large diameter afferent neuropathy (complete loss of proprioception) are unable to stand or walk without vision (148, 149). Sway-referencing the support surface to the postural oscillations, however, has mechanical consequences that must be taken into account when interpreting the standing balance behavior to this modified ankle somatosensory feedback. Because the ankle joint angle remains relatively constant as the body oscillates back and forth, minimal deformation of the ankle tissues (muscles, tendons, ligaments, skin) occurs. This prevents the development of length and velocity dependent passive forces that normally contribute to the stabilizing torque required to remain upright (17). Considering that passive forces are estimated to contribute between 10 and 90% of the net torque required to stand (16, 17, 20, 150), it is not clear what portion of the postural stability changes observed during sway-referencing are due to the contribution of ankle somatosensors versus the modulation of the active component of standing to compensate for a reduction in passive forces contributing to standing. A potential approach to explore these possibilities could involve simulating/altering the muscle activation to muscle torque transfer functions using robotic devices replicating the control of standing balance (13, 151, 152) (see Figure 2B and Mechanical and Sensory Approaches).

The isolated contribution of somatosensory cues to standing balance has been assessed using balance control of a body-equivalent load (8). Participants supported by a rigid frame with their head immobile (minimizing visual and vestibular cues) balanced a load with their feet that mimics the dynamics of an inverted pendulum (8, 130, 146, 153–155) (see Figure 3B). To distinguish contributions from muscle proprioceptors and foot sole cutaneous cues, skin receptors have been minimized by cooling or anesthetizing the feet (153, 156). The general consensus from these experiments is that ankle muscle receptors provide adequate inputs for maintaining standing balance. Although the range and variability of the body-equivalent load oscillations were larger than for natural standing balance (where all cues are available), participants could stabilize the load with only cues from the ankle muscle proprioceptors (153). The similar frequency characteristics of “whole-body” sway between these conditions further supported the conclusion that ankle muscle receptors are sufficient to maintain standing balance. Confirmatory findings by other groups provide additional validation regarding this conclusion (147, 157).

Additional somatosensory information may be incorporated within the balance control loop through the use of light touch. When subjects make light contact with a stationary external reference—typically with a finger—postural sway is reduced despite the negligible mechanical stabilizing effect of touch (158, 159). This suggests that cues encoded from low contact forces are incorporated as a sensory signal contributing to the balance control loop. Improved standing balance stability has also been observed when two standing subjects make light finger contact with one another (160–162). Using a simple modeling approach, Reynolds and Osler (162) suggested that interpersonal contact while standing is beneficial even if the balance controller does not distinguish self and partner motion. Taken together, these studies highlight the potential for light touch to alter sensory feedback within the balance control loop.

### Visual cues

A simple method to manipulate visual cues is to have subjects stand with the eyes closed or in the dark. Compared to eyes open, eyes closed (or darkness) increases quiet whole-body oscillations (9, 38, 163–165), but the low frequency components require long sampling durations of stance (>300s) to be captured accurately (14). The importance of visual information for standing has also been revealed by manipulating the number of fixation targets (166, 167), type of lighting (168) and depth cues (169–171). In a series of experiments, Paulus et al. (38) reported increases in postural stability under conditions with improved visual acuity, increased area of the central visual field and increased retinal displacement (caused by decreasing the eye-object distance). These observations emphasize that the influence of vision on standing balance is dependent on the features of the visual scene. An alternative approach is to keep visual signals constant on the retina (effectively sway-referencing vision) by having participants view a scene that moves according to the motion of the whole-body (144, 145, 154, 172). Under these conditions, balance was more unstable compared to when the eyes were closed (145, 173, 174). McCollum et al. (174) rationalized that this occurs because in the visual sway-referenced condition, there is a central integration conflict (or mismatch) between different sensory channels (i.e., vision-vestibular, vision-somatosensory). Collectively, these studies suggest that visual cues contribute to standing balance, and are likely fused with other signals encoding whole-body with postural oscillations.

**Figure 4:**
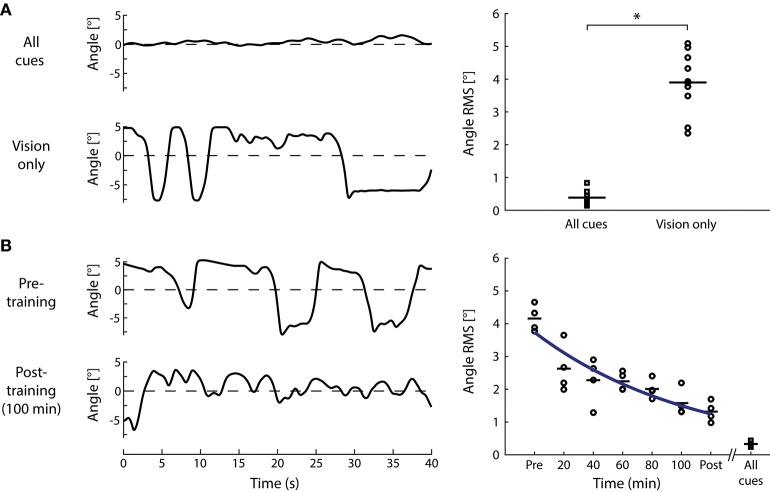
Standing balance using only visual cues. **(A)** Subjects stood in a robotic balance simulator while either all sensory cues (see Figure 3A) or only visual cues of balance were provided (see Figure 3C). Angular oscillations of a representative subject (left) when balancing with all cues or only vision show an increase in angular sway when balancing with only vision. Mean removed root-mean-square (RMS) of angular oscillations of all subjects tested (*n* = 10, right) exhibited the same increase when using visual cues to balance (paired *t*-test; *t*_9_ = −13.2, *P* < 0.001). Squares represent the “all cues” condition and circles represent the “vision only” condition. **(B)** Four of the original 10 subjects subsequently went through 5 days of training (~20 min per day) under the vision only condition. Angular oscillations of a representative subject (left) show a decrease after 100 min (5 days) of training compared to the pre-training vision only condition. Mean-removed RMS of angular oscillations progressively decreased with each session of training (circles), but always remained above the all cues conditions (squares). For illustrative purposes, the blue line shows the fitting of mean angle RMS to an exponential function (*y* = 4.4893 *e*^−0.1829 *x*^) using a least-square method. In both graphs, horizontal lines represent the mean of standard oscillation across all subjects and *P < 0.001.

An alternative approach to determine the role of visual cues in standing balance involves determining if these cues alone are sufficient to remain upright (see Figure 3C). Nagata et al. (157) devised a computer-controlled inverted pendulum allowing participants to apply forces and moments to the ground but experiencing only the visual consequences of their motion. Participants were stable in space while a motor replicated the visual signals of balance according to their motor actions—hence subjects attempted to balance an equivalent body load with sensory feedback limited mostly to visual cues (others included somatosensory cues of feet pressure changes and muscle contractions). Nagata et al. (157) reported that vision only contributed to the reduction of sway below 0.4 Hz. This aligns with previous suggestions that vision may primarily contribute to the low frequency (<1 Hz) control of standing balance (170, 175, 176). Although visual perturbations can evoke sway behavior as high as ~2 Hz (20), responses tend to decline rapidly above 0.8 Hz. Nagata et al. (157) argued that the processing of visual information was too slow such that vision provided only a minor influence on the control of standing balance. A limiting factor of their approach, however, was that the rotational axis of the visual enclosure was not collinear with the ankle joints (154). Loram and colleagues, in contrast, have shown that participants standing braced can balance a real or virtual inverted pendulum with similar mechanics of the standing body using their hand to move a spring or a joystick with only visual cues of motion (155, 177, 178). To address this apparent discrepancy on the role of visual cues to maintain standing balance, we performed a simple experiment. Ten healthy subjects participated in this study after giving their written informed consent. The experiment protocol conformed to the Declaration of Helsinki and was approved by the University of British Columbia's Clinical Research Ethics Board. Similar to Fukuoka et al. (154), braced upright participants balanced with the expected visual cues of self-motion programmed to replicate the motion of an inverted pendulum in the antero-posterior direction (see Figure 3C). Initially, all participants (*n* = 10) exhibited difficulties in keeping the visual cues of motion within the balance limits (i.e., 6° anterior and 3° posterior). Sway variability was 5–6 times larger than when balancing a robotic simulator using all sensory cues (Figure 4A). After 5 days of training to balance with only visual cues of motion (~20 min per day), their ability to balance within the programmed limits improved substantially (Figure 4B). Participants exhibited a ~75% decrease in sway variability but this variability remained twice that observed when balancing with all sensory cues. These data show that although subjects exhibit initial difficulties in balancing with only visual cues of motion, they can adapt and use these cues to control standing balance with practice.

### Vestibular cues

The contribution of vestibular cues has been inferred by assessing the standing balance behavior while carefully controlling the available cues to remain upright. One approach involved characterizing postural oscillations while sway-referencing vision as well as the support surface (145, 174). By minimizing visual and ankle somatosensory cues, Nashner and colleagues were targeting the role of vestibular signals in maintaining upright stability. Participants exhibited difficulty in maintaining upright posture when vision and ankle proprioception were sway referenced, sometimes experiencing falls (145, 174). However, the limitation discussed above regarding the lack of passive forces contributing to upright stability under the sway-referencing of the support surface also applies to these experiments. A second approach consisted of comparing postural oscillations during normal upright stance (including vestibular cues) and during balancing a body-equivalent load while braced (excluding vestibular cues). When limiting whole-body movements to the ankle joints in both conditions, Fitzpatrick et al. (153) showed that balance stability was similar if vestibular cues contributed to the control of balance or not, irrespective of visual cues. Altogether, observations from these two distinct approaches suggest that vestibular cues provide limited benefit over visual and somatosensory cues to the control of standing balance. In support of this idea, vestibular loss patients can maintain upright stance with vision and somatosensory cues (even at the onset of the deficit), and over time, the instability is reduced due to compensation processes (179–182) and possibly from neural adaptation such as that observed in non-human primates (183–185).

Ongoing manipulation of vestibular cues according to postural oscillations was assessed in a different context by Héroux et al. (186). Participants were standing on foam with eyes closed while exposed to an electrical vestibular stimulus. The electrical stimulus was designed to replicate the general dynamics of primary semicircular afferents modulations during standing balance and coupled in real-time to the recorded movements of the head (see Figure 2B). Conceptually, this head-coupled vestibular stimulus increased or decreased vestibular gain depending on the polarity of the stimulus with respect to the measured head motion. When the stimulus was applied, postural oscillations increased 4-fold. This finding bears some similarity to the decreased postural stability observed in acute unilateral vestibular loss patients (181, 187, 188) who are faced with asymmetric vestibular inputs. Although these results suggest that altering the gain of vestibular cues during standing influence the balance behavior, additional work is needed to determine if such vestibular cues of standing delivered in isolation (i.e., standing fixed to a rigid backboard) are sufficient to allow humans to balance upright.

## Combining manipulations targeting the ongoing control of quiet standing balance with imposed external perturbations

Although manipulations targeting the ongoing control of standing balance can indicate limits of adaptability in the controller, there are limitations with interpreting standing behavior (forces, torques, sway) when sensory cues are manipulated in isolation. Specifically, while the combination of sensory cues can be well controlled, manipulations of the ongoing control of balance do not provide a known external perturbation signal. Van der Kooij et al. (57) compared different approaches to assess standing balance and showed that an external perturbation is needed to characterize the mechanisms governing balance. Applying imposed perturbations while controlling specific parameters of the ongoing control of standing combines the strengths of both approaches, affording a unique opportunity to reveal operating principles of the balance system and potentially revealing some of its inherent limitations. In the following section, we describe how imposed perturbations during well-controlled sensory manipulations have revealed fundamental features of standing balance such as inter-sensory interactions and re-calibration of sensory feedback loops. This includes the use of mechanical and robotic balance systems that allow for the replication of standing balance dynamics and provide users full control to virtualize parameters of the balance task. Finally, we briefly present an approach to alter the vestibular contribution to standing and discuss the resulting adaptation occurring in the control of standing balance.

### Mechanical and sensory approaches

Pioneering work using a combination of perturbation approaches was conducted by Fitzpatrick et al. (130). The authors used their whole-body equivalent load device to explore how the vestibular control of standing balance—characterized with EVS-evoked muscle responses—was modulated by the sensory cues contributing to postural stability. Fitzpatrick et al. (130) revealed strong context-dependency of the vestibular control of standing: vestibular-evoked muscle responses were absent when subjects balanced the body-equivalent load using only somatosensory cues. This suggests that although lower limb somatosensory cues are sufficient to maintain upright stance, balance-relevant vestibular feedback is required to engage the response to an external vestibular perturbation signal (130).

Cenciarini and Peterka (81) combined support surface perturbations (pseudorandom ankle tilt stimuli and sway-referenced conditions) with step EVS pulses to test predictions from their sensory re-weighting hypothesis (20). The authors showed that the amplitude of vestibular-evoked whole-body responses increased when concomitant perturbations were applied to the support surface and were largest when the ankle joint was sway-referenced. These observations corresponded well with predictions from their computational model and were interpreted as providing support for the sensory re-weighting hypothesis (79, 80, 189, 190). In this case, the limited balance feedback from the ankle proprioceptors during sway-referencing was interpreted as requiring an increased contribution of vestibular signals for standing balance as reflected by the larger EVS-evoked responses. Note that, as similarly discussed in section Vestibular Perturbations, support surface perturbations and sensorimotor manipulations may influence balance stability which in turn modulates vestibular-evoked balance responses (138, 139). Carefully designed experiments are needed to determine the relative contribution of standing balance state (i.e., angular position and angular velocity) and sensory re-weighting on the modulation of vestibular-evoked balance responses.

To take advantage of the possibilities enabled by manipulations of the balance control loop, our group developed a robotic system that can replicate and/or modify specific parameters of the sensorimotor control of standing balance (Figure 2B) (151). Upright participants are braced to a rigid backboard mounted atop a six-degree of freedom Stewart mechanism. Through a computer simulation in which the mechanics, sensory feedback and environment of standing can be simulated or altered, the robot rotates the whole-body about the ankle joints based on the real-time ground reaction forces and moments applied by the participants. Motion of the robot can be restricted to the anterior-posterior direction and the force plates are mounted to an ankle-tilt platform, allowing independent control of whole-body and ankle movements (191). When programmed to simulate an inverted pendulum, movement of the subjects actuating the robot replicates the torque-angle relationship of the whole-body during unrestricted standing balance (13). Under these subject-in-the-balance-loop conditions, a plantar-flexor torque is necessary to maintain the body in a forward leaning position.

Using this robotic balance simulator, Luu et al. (131) revisited the hypothesis that balance-relevant vestibular feedback is required to engage the response to an external vestibular perturbation signal. First, Luu et al. (131) showed that vestibular feedback (whole-body sway) independent from the balance task was not sufficient to elicit muscle responses to vestibular stimuli. Forbes et al. (139) complemented these findings by allowing participants to balance only along one plane (anteroposterior or mediolateral) while controlling the orientation of the head—and the direction of the vestibular-induced error signal. As the direction of balance and that of the vestibular error signal rotate orthogonally to one another, vestibular-evoked muscle responses are progressively suppressed even though subjects are engaged in balance. Hence, the vestibular contribution to balance muscle activity depends not only on the contribution of vestibular feedback to the ongoing muscle activity but also on the cross-product of the direction of balance instability and the direction of the induced vestibular error. Second, Luu et al. (131) addressed the possibility that balance-relevant vestibular signals must be temporally and spatially coupled to the motor commands to engage the vestibular control of standing. Participants stood atop the robotic balance system under two conditions: (1) with coupled sensory and motor signals, where subjects actively controlled the motion of their body in space by modulating their ankle torques (replicating normal standing), and (2) with decoupled sensory and motor signals, where the robot imperceptibly took control and imposed whole-body motion to the subjects following a pre-determined trajectory independent of their ankle torques. For the latter condition, subjects continued to actively modulate their ankle torques despite them not influencing the motion of their body, thus resulting in a discrepancy between predicted and actual sensory feedback associated with the standing balance task. Despite subjects demonstrating poor conscious awareness of the transitions between these two conditions (i.e., self vs. robot-controlled whole-body motion), vestibular-evoked muscle responses were attenuated when motor and sensory cues of balance were decoupled. These observations suggest that congruency between predicted and actual sensory signals is required to engage the vestibular control of standing balance. One caveat to these observations, however, is that the congruency of multiple balance feedback cues (visual, vestibular, somatosensory) was manipulated simultaneously (i.e., either all congruent or none were congruent). Hence, it remains unclear how individual sensory cues interact with the balance responses to vestibular error signals.

Forbes et al. (139) further used the robotic balance system to explore the adaptability of the control of standing balance. They modified the balance simulation by reversing the direction of whole-body motion produced by the measured ankle torques, effectively inverting the roles of the muscles controlling balance in the anteroposterior plane. Subjects were instructed to close their eyes and the ankle-torque relationship was maintained, mainly targeting the reversal to vestibular feedback. Under these reversed conditions, a dorsi-flexor torque is necessary to maintain the body in a forward leaning position. Participants adapted within 30–90 s to the reversed balance control. When EVS was applied, subjects swayed in the same direction for both the control and reversed balance conditions. To induce the same whole-body movement, the motor outputs from the balance controller (e.g., torque and muscle responses), however, were reversed and delayed. This indicates that the neural centers controlling standing balance can rapidly integrate the state of the relationship between motor commands and whole-body sensory feedback, and generate appropriate muscle responses to correct for the induced vestibular error signals. Such swift re-associations of sensorimotor relationships may reflect our flexibility to maintain bipedal postures in varied settings, like when stepping from shore onto a stand-up paddle board. Similar reversals of vestibulomotor responses have been observed in the vestibulo-ocular reflex (VOR) during exposure to optical reversals of vision, although adaptation typically required days or weeks to fully invert vestibular-evoked eye movements (192–194). Despite the temporal differences in the balance and VOR adaptation to the reversals, the detailed characterization of the cellular mechanisms in the cerebellum and vestibular nuclei involved in the plasticity of the VOR [see review by Cullen and Mitchell (195)] may point toward similar neurophysiological processes playing a role in vestibulomotor adaptations for balance. In non-human primates, adaptations in neuronal recordings of vestibular nuclei and cerebellar neurons have been observed on a trial-by-trial basis (196). Over exposure to a novel relationship between motor commands and consequent head movement (altered head-neck dynamics), neuronal responses adapt from encoding head motion as externally generated to one that is self-generated. The multisensory convergence of sensory afferents at the vestibular nuclei and their projections to descending spinal tracts (197, 198) suggest that the vestibular nuclei contribute to the adaptive mechanisms observed in the vestibular control of balance.

### Sensory and sensory approaches

Carefully manipulating the information from multiple sensory inputs further allows one to explore inter-sensory interactions in standing balance. Several groups have investigated how varying the availability and quality of visual cues interacts with the vestibular-evoked balance response to EVS (129, 130, 171, 199, 200). Day and Guerraz (171) manipulated the quality of visual cues providing information regarding whole-body oscillations during standing balance. Participants stood in a dark room while viewing nothing, a single light-emitting diode, a two-dimensional array of light-emitting diodes or a three-dimensional array of light-emitting diodes. The authors probed the vestibular control of balance using EVS under these different conditions to determine how the structure of visual cues related to standing balance influenced vestibular-evoked responses. In healthy controls, they showed that the early parts of vestibular-evoked responses vary when pre-stimulus visual information differs (i.e., light or dark), even when the post-stimulus feedback visual environments are equivalent. Feedback effects from the post-stimulus environment were also observed, affecting the later parts of the balance response (>~400 ms). This setting of the vestibular channel's gain can explain how vestibular responses evoked in healthy controls change with the amount of available visual cues.

Mian and Day (138) explored how sensory information derived from light touch can influence the direction of the vestibular-evoked balance response. Standing subjects were probed with EVS while lightly touching a stationary flat surface aligned laterally to the subjects. Despite light touch providing negligible mechanical stabilizing effects on the body, the response to EVS was biased toward the anteroposterior direction. As sensory cues from light touch are thought to be transformed into ongoing proprioceptive feedback for standing balance, this suggests that the gain of the vestibular-evoked balance response is spatially-modulated by the orientation (or direction) of balance-relevant proprioceptive feedback. Careful interpretation of these findings is warranted because light touch also reduced whole-body sway in the mediolateral plane.

As stated above, Héroux et al. (186) designed biologically-plausible head-coupled electrical vestibular stimuli to manipulate vestibular gain in healthy volunteers standing upright on foam with eyes closed. While balance oscillations increased four-fold when the electrical stimuli were applied (some subjects needed support to avoid a fall), the amplitude of the vestibular-evoked muscle responses (probed with an independent low-amplitude EVS signal) decreased. The authors further evaluated whether the participants could adapt to ongoing modulation of the vestibular cues associated with standing balance. The critical concept here was to determine if an imposed vestibular error signal that is coupled to the ongoing control of quiet standing balance can be calibrated and incorporated in the balance control loop. Participants were exposed to a re-calibration period of 240 s where the in-the-loop modified vestibular cues were provided with no foam and/or eyes open. Following this period, participants could maintain standing balance (on foam with eyes closed): postural sway and vestibulomotor response amplitudes returned to baseline. These results could not be explained by a down-regulation (or reweighting) of vestibular cues because matching levels of EVS that were uncoupled from head motion (hence remained an external imposed perturbation) did not yield any adaptation following a 240s re-calibration period. Instead, these observations indicate that the balance controller can integrate an external vestibular error signal into its control loop and likely interpret it as a self-generated signal as long as that signal follows the expected sensory dynamics encoding ongoing quiet standing balance. Consequently, a vestibular signal that was deemed an error signal before re-calibration was transformed into a meaningful signal that was used to maintain upright balance.

## Future directions

Sensorimotor manipulations of the balance control loop can target how muscle activation is related to the ground reaction forces and moments acting on the subject as well as the sensory feedback experienced (perceived or not) by participants maintaining standing balance. Critical questions to address include determining the influence of the state of standing balance stability [see (138, 139)] on imposed perturbations, how sensory signals are used to control standing balance under challenging conditions along with the limits of our capability to maintain upright stance. As a specific example, Luu et al. (131) proposed that a spatial and temporal relationship between sensory and motor signals is required to engage the vestibular control of standing balance. The factors underlying this spatio-temporal relationship need to be explored as well as their influence on our capability to remain upright. Future experiments should also target how imposed visual perturbations are integrated in the control of standing balance under manipulations similar to those explored using imposed electrical vestibular stimuli (131, 138, 139) to determine if previous findings can be generalized and truly reflect fundamental mechanisms of the balance control loop. Building on the work from Héroux et al. (186), it is also conceivable to imagine innovative ways to characterize the unique contribution of sensory cues to the control of standing balance. As we learn more about the dynamics of standing and the resulting code from specific sensory afferents, artificial stimuli can be envisioned to replicate the neural code and assess its contribution to standing. For example, knowledge regarding the firing behavior of muscle spindle afferents during upright stance would permit the creation of a range of stimuli (intraneural electrical, mechanical or miniaturized robotics) to mimic it. The keys to such endeavors include a better understanding of the physiological code underlying standing balance and concerted efforts to replicate it during well-controlled balance-relevant experiments.

## Conclusions

We have reviewed externally imposed perturbations and manipulations of the balance control loop that can be used to reveal the multisensory cue integration, task-dependent sensory processing and sensorimotor adaptation underlying the control of standing balance. We presented imposed external perturbations that elicit postural responses when the stimulus is related to the context of standing balance. These balance-specific approaches can provide important insight on the factors influencing the control of standing balance. We also described manipulations of the balance control loop which allow for the modification of mechanical and/or sensory dynamics to target the ongoing control of standing balance. Finally, we presented how combining imposed perturbations and manipulations of the balance control loop, including robotics and sensory manipulations, can reveal important principles underlying the maintenance of standing balance such as spatio-temporal congruency between sensory and motor signals, rapid re-association of sensorimotor relationships and re-calibration of vestibular signals in the balance control loop. We reason that by carefully considering the neural code of quiet standing, well-controlled experiments can utilize these combined imposed perturbations and manipulations of the balance control loop approaches to uncover the fundamental mechanisms of balance control.

## Author contributions

BR, PAF, and J-SB contributed to conception and design of the review. RT collected and analyzed the data. BR and J-SB wrote the first draft of the manuscript. All authors contributed to manuscript revisions, and read and approved the submitted version.

### Conflict of interest statement

The authors declare that the research was conducted in the absence of any commercial or financial relationships that could be construed as a potential conflict of interest.
